# Ovarian Cancer Relies on Glucose Transporter 1 to Fuel Glycolysis and Growth: Anti-Tumor Activity of BAY-876

**DOI:** 10.3390/cancers11010033

**Published:** 2018-12-31

**Authors:** Yibao Ma, Wei Wang, Michael O. Idowu, Unsong Oh, Xiang-Yang Wang, Sarah M. Temkin, Xianjun Fang

**Affiliations:** 1 Department of Biochemistry & Molecular Biology, Virginia Commonwealth University School of Medicine, 1101 East Marshall Street, Richmond, VA 23298, USA; yibao.ma@vcuhealth.org (Y.M.); wei.wang1@vcuhealth.org (W.W.); 2 Pathology, Virginia Commonwealth University School of Medicine, Richmond, VA 23298, USA; michael.idowu@vcuhealth.org; 3 Neurology, Virginia Commonwealth University School of Medicine, Richmond, VA 23298, USA; unsong.oh@vcuhealth.org; 4 Human & Molecular Genetics, Virginia Commonwealth University School of Medicine, Richmond, VA 23298, USA; xiang-yang.wang@vcuhealth.org; 5 Gynecological Oncology, Virginia Commonwealth University School of Medicine, Richmond, VA 23298, USA; sarah.temkin@vcuhealth.org

**Keywords:** ovarian cancer, glucose transporter 1, BAY-876, glycolysis, patient-derived xenograft

## Abstract

The recent progresses in understanding of cancer glycolytic phenotype have offered new strategies to manage ovarian cancer and other malignancies. However, therapeutic targeting of glycolysis to treat cancer remains unsuccessful due to complex mechanisms of tumor glycolysis and the lack of selective, potent and safe glycolytic inhibitors. Recently, BAY-876 was identified as a new-generation inhibitor of glucose transporter 1 (GLUT1), a GLUT isoform commonly overexpressed but functionally poorly defined in ovarian cancer. Notably, BAY-876 has not been evaluated in any cell or preclinical animal models since its discovery. We herein took advantage of BAY-876 and molecular approaches to study GLUT1 regulation, targetability, and functional relevance to cancer glycolysis. The anti-tumor activity of BAY-876 was evaluated with ovarian cancer cell line- and patient-derived xenograft (PDX) models. Our results show that inhibition of GLUT1 is sufficient to block basal and stress-regulated glycolysis, and anchorage-dependent and independent growth of ovarian cancer cells. BAY-876 dramatically inhibits tumorigenicity of both cell line-derived xenografts and PDXs. These studies provide direct evidence that GLUT1 is causally linked to the glycolytic phenotype in ovarian cancer. BAY-876 is a potent blocker of GLUT1 activity, glycolytic metabolism and ovarian cancer growth, holding promise as a novel glycolysis-targeted anti-cancer agent.

## 1. Introduction

Ovarian cancer is the most lethal of gynecological malignancies. The dismal prognosis results from the inability to detect the disease at an early, curable stage and the lack of effective therapies for recurrent disease which inevitably develops chemotherapy resistance [1]. Recent advances in understanding of metabolic transformations have opened new and promising avenues for developing alternative therapies to target cancer cell metabolism for ovarian cancer and other malignant diseases [2]. The most prominent aspect of metabolic transformation is the glycolytic phenotype or the Warburg effect, whereby cancer cells sustain a high rate of aerobic glycolysis [3]. Substantial evidence supports the necessity for hyperactive glycolysis in rapidly dividing tumor cells [4]. The process provides not only a quick way to generate ATP but also metabolic intermediates for biosynthesis of complex macromolecules such as amino acids and lipids. Indeed, inhibitors of glycolytic enzymes such as hexokinase 2 (HK2) [5,6], phosphofructokinase 2 (PFK2) [7], pyruvate kinase M2 (PKM2) [8], lactate dehydrogenase A (LDHA) [9], and pyruvate dehydrogenase kinase (PDK) [10] show anti-tumor effects. However, clinical trial failures with the HK2 inhibitors 2-deoxyglucose and lonidamine have cast doubt on the feasibility of glycolysis-targeted therapies [11]. Apparently, a more comprehensive understanding of functionally relevant mediators of tumor glycolysis and development of their specific inhibitors are critical to the success of anti-glycolysis strategy.

In addition to glycolytic enzymes, glucose transporters on plasma membranes facilitate glucose uptake. There are two classes of glucose transporters, sodium-dependent glucose transporter (SGLT) and facilitative glucose transporter (GLUT) [12]. Various members of the GLUT family, especially GLUT1-4 are reported to be upregulated in cancer [13]. For example, GLUT2 is more markedly elevated than other GLUTs in hepatocellular carcinoma, and a strong expression of GLUT2 correlates with poor overall patient survival [14]. In bladder cancer and glioblastoma, GLUT3 was found to be overexpressed in more invasive tumors than lower-grade ones [15,16]. Over-expression of the insulin-independent GLUT1 seems to be most common among different types of cancer [17]. These include colorectal carcinoma, lung adenocarcinoma, breast carcinoma, ovarian carcinoma, squamous cell carcinoma, and glioblastoma [13]. The activity of GLUT1 is regulated not only by its expression level but also by protein trafficking to the plasma membrane. Activities of hypoxia-inducible factor 1 (HIF1) [18], c-Myc [19], Ras [20] and Akt [21] and mutation of the tumor suppressor p53 [22] have been linked to overexpression or membrane location of GLUT1.

Although substantial studies support abnormal GLUT1 expression/activity which correlates with glycolytic metabolism in cancer [21,23,24,25], the exact oncogenic function of GLUT1 remains obscure. Especially in ovarian cancer, there is little direct evidence for a causal role of GLUT1 in the development or maintenance of tumor glycolytic phenotype [24,25]. The question is complicated by potential involvement of many other glycolytic enzymes that are also often dysregulated in ovarian cancer. Moreover, functional redundancy or metabolic compensation from other isoforms of the large GLUT family could prevent or minimize the dependence of glucose supply on a single GLUT member. Thus, the availability of highly specific GLUT1 inhibitors is critically important to interrogate the function of GLUT1 in cancer glycolysis and its potential as an oncotarget.

Among small-molecule GLUT1 inhibitors [23,26,27,28,29,30,31,32], STF-31 [23] and WZB117 [26] were reported to inhibit GLUT1-mediated glucose uptake at low µM concentrations and to show some anti-cancer activities in xenograft tumor models. However, the GLUT1 selectivity and potencies of these earlier GLUT1 inhibitors are controversial [33,34]. Recently, BAY-876 [*N*^4^-[1-(4-cyanobenzyl)-5-methyl-3-(trifluoromethyl)-1*H*-pyrazol-4-yl]-7-fluoroquinoline-2,4-dicarboxamide] was identified as a most promising GLUT1 inhibitor [35]. BAY-876 is unique in its single-digital nanomolar potency and a selectivity factor of >100 against GLUT2, GLUT3, and GLUT4. It also shows ideal metabolic profiles in vitro and high oral bioavailability in vivo [36]. However, the applicability of BAY-876 to cancer intervention is largely unknown. Since its discovery in 2016, preliminary anti-tumor activity of BAY-876 has been reported only in an abstract containing no information on drug dose, duration of treatment and animal tolerance [36].

In this study, we have taken advantage of this new GLUT1 candidate inhibitor along with molecular approach to examine the roles of GLUT1 in glycolysis regulation and growth of ovarian cancer cell lines and ovarian cancer patient-derived xenografts (PDXs). Our results provide the first direct evidence that GLUT1 is indispensable for both basal and induced glycolysis in ovarian cancer cells. We validate GLUT1 as an effective anti-cancer target by demonstrating that BAY-876 blocks GLUT1-dependent glycolytic metabolism in ovarian cancer, resulting in potent inhibition of tumor cell growth in vitro and in vivo.

## 2. Results

### 2.1. GLUT1 Is Overexpressed and Required for Maintaining Active Glycolysis in Ovarian Cancer

To gain an insight into potential dysregulation of GLUT isoforms in ovarian cancer, we analyzed the Oncomine cancer profiling database [37]. Most of these studies available on Oncomine clearly demonstrated that *GLUT1* mRNA was significantly increased in high-grade serous ovarian carcinoma, the most common form of epithelial ovarian cancer (Supplementary Figure S1A). Based on the only available Hendrix Ovarian Dataset, *GLUT1* mRNA was also overexpressed in ovarian clear cell, endometrioid, and mucinous carcinomas [38] (Supplementary Figure S1A). In contrast to *GLUT1*, expression of *GLUT2*, *3*, or *4* in ovarian cancer did not show consistent changes among different studies available on Oncomine. Mechanistically, *GLUT1* overexpression seems to be linked to its gene amplification as evidenced by the TCGA Ovarian 2 Dataset that compared 130 normal ovaries with 607 serous ovarian cystadenocarcinomas (Supplementary Figure S1B).

**Figure 1:**
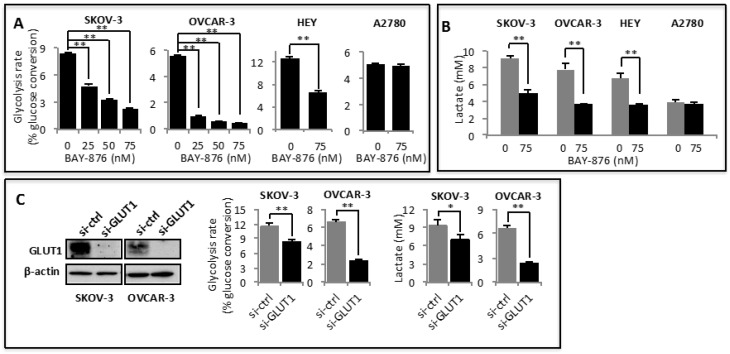
Glucose transporter 1 (GLUT1) activity is required for maintenance of hyperactive glycolysis in ovarian cancer. Ovarian cancer cell lines were treated for 24 h with indicated concentrations of BAY-876. The glycolytic rate (**A**) and lactate concentrations in supernatants (**B**) were measured as described in Materials and Methods. The lactate levels are presented as mM concentrations present in 1 mL culture supernatants normalized to 5 × 10^5^ cells over 24 h incubation. In (**C**) GLUT1 was silenced by siRNA in SKOV-3 and OVCAR-3 cells and effects of siRNA knockdown on glycolysis and lactate production were determined. For this and all the following figures, the data presented were mean ± SD of triplicates, representative of 3 independent experiments unless otherwise indicated. * *p* < 0.05; ** *p* < 0.01.

To understand the biological outcomes of GLUT1 dysregulation in oncogenesis, we asked if ovarian cancer cells rely on GLUT1 to support glycolytic metabolism. As we and others have demonstrated previously [39,40], ovarian cancer cells exhibited high basal glycolytic activity in regular culture conditions (Figure 1A). Although BAY-876 has been shown to inhibit glucose uptake (35,36), its effect on the downstream glycolytic metabolism is not, known. We thus examined the effects of BAY-876 on glycolysis and lactate production in ovarian cancer cell lines including A2780 known to lack a functional GLUT1 [41] as a negative control. As shown in Figure 1A, incubation with BAY-876 dose-dependently decreased glycolytic rates in SKOV-3, OVCAR-3, and HEY cells. Similarly, BAY-876 reduced lactate levels present in culture supernatants of these cells (Figure 1B). Although this anti-glycolytic effect of BAY-876 was detectable at single-digital nanomolar concentrations, half-maximum suppression was achieved with 25–50 nM of the compound. We also observed similar anti-glycolytic activity of BAY-876 in other commonly used ovarian cancer cell lines such as OVCR-429 and OVCA-432.

In the A2780 ovarian cancer cell line, GLUT1 was reported to co-localize with mutant PTEN at the nucleus instead of the plasma membrane [41]. Consistent with BAY-876 suppressing glycolysis via specific inhibition of GLUT1 rather than other GLUT members or off-target proteins, BAY-876 did not affect glycolysis or lactate production in A2780 cells (Figure 1A,B). The specific effect of BAY-876 on GLUT1-mediated glycolysis was further confirmed by siRNA silencing of *GLUT1*. The molecular approach decreased glycolysis rate and lactate production in SKOV-3 and OVCAR-3 cells (Figure 1C).

### 2.2. GLUT1 Mediates Upregulation of Glycolysis in Stress Conditions

**Figure 2:**
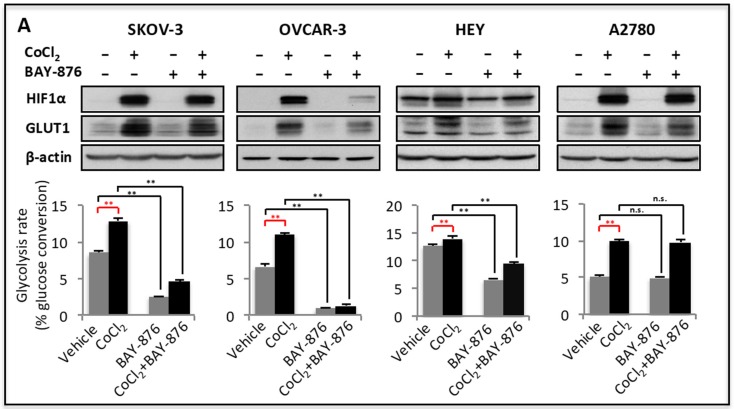

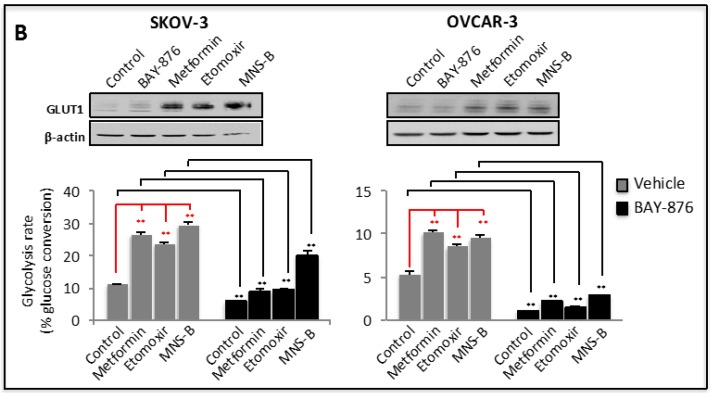
GLUT1 mediates hypoxia- and energy stress-induced glycolysis. Ovarian cancer cell lines were treated for 24 h with CoCl_2_ (150 µM) in the presence or absence of BAY-876 (75 nM) (**A**). In (**B)**, Ovarian cancer cell lines were treated for 24 h with vehicle, metformin (2 mM), etomoxir (0.25 mM), or MNS-B (50 nM) in the absence or presence of BAY-876 (75 nM). Expression of GLUT1 and HIF-1α proteins was analyzed by immunoblotting. Glycolytic rates were quantified as in Figure 1. The statistical significances of glycolytic induction and BAY-876-dependent inhibition were indicated with red and black asterisks, respectively. n.s.: * *p <* 0.05; ** *p* < 0.01.

The basal aerobic glycolysis is generally high in ovarian cancer and other malignant cells compared to their normal counterparts. The glycolytic activity of cancer cells also fluctuates in response to tumor microenvironmental cues to meet the bioenergetic and biosynthetic needs of rapidly growing tumor cells. In particular, glycolysis is heightened in response to hypoxic conditions [42]. *GLUT1* is known to be one of hypoxia target genes, upregulated by HIF [18]. We used cobalt chloride (CoCl_2_) as a means of stabilizing HIF to examine GLUT1 inducibility in ovarian cancer cells. As shown in Figure 2A, GLUT1 was highly induced by CoCl_2_ in all ovarian cancer cell lines. The induction of GLUT1 was associated with a rise in cellular glycolysis (Figure 2A). BAY-876 reduced both basal and CoCl_2_-stimulated glycolysis in SKOV-3, OVCAR-3 and HEY cells. Although CoCl_2_ increased GLUT1 expression and glycolysis in A2780 cells, BAY-876 had no effects on either basal or CoCl_2_-driven glycolysis, suggesting that GLUT1-independent mechanisms are involved in HIF-driven glycolysis in A2780 cells.

Interference with electron transport chain (ETC) augmented glycolytic metabolism likely as an adaptive response to bioenergetic loss [43]. We therefore hypothesized that GLUT1 could mediate the glycolytic response to these bioenergetic stress conditions. Several known membrane-permeable Complex I inhibitors such as metformin [44], etomoxir [45], and manassantin B (MNS-B) [43] were tested for their effects on GLUT1 expression and glycolysis. Each of these compounds induced GLUT1 protein expression and concomitantly enhanced glycolytic rates in SKOV-3 and OVCAR-3 cells (Figure 2B). In the presence of BAY-876, the glycolytic induction was dramatically blunted (Figure 2B). Together, these results indicate that GLUT1 acts as a critical mediator of both basal and stress-induced glycolysis in ovarian cancer cells.

### 2.3. Inhibition of GLUT1 Causes Bioenergetic Loss and Activation of AMP-Activated Protein Kinase (AMPK)

**Figure 3:**
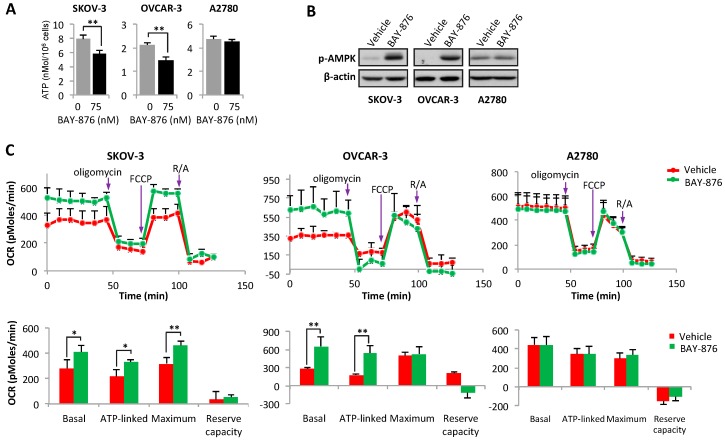
Inhibition of GLUT1 decreases ATP, activates AMPK and enhances OCR. SKOV-3, OVCAR-3 and A2780 cells were treated with vehicle or BAY-876 (75 nM). Cellular ATP contents (nMol of ATP in 10^6^ cells) (**A**), and levels of phosphorylated AMPKα (**B**) were determined with an ATP bioluminescence assay and immunoblotting analysis, respectively. In (**C**), OCR in these cell lines treated with vehicle or BAY-876 (75 nM) was measured with the Seahorse XF24 Analyzer as detailed in Materials and Methods. The background in cell-free wells was subtracted from readings of all other wells. The presented in upper panel were real time OCRs (pMoles/min, mean ± SD of triplicate assay) over the course of measurement. In lower panel, basal mitochondrial respiration (Basal), ATP-linked respiration (ATP-linked), maximal respiratory capacity (Maximum) and Reserve capacity were calculated and statistically analyzed to show the effects of BAY-876. * *p* < 0.05; ** *p* < 0.01.

Unlike normal cells that use oxidative phosphorylation as a major bioenergetic source, cancer cells rely heavily on glycolysis for substrate-based phosphorylation of ADP to form ATP [44]. We next determined whether GLUT1 inhibition is sufficient to reduce ATP production in ovarian cancer cells. As shown in Figure 3A, cellular ATP levels were significantly decreased by treatment of SKOV-3 and OVCAR-3 with BAY-876. In agreement with decreased ATP abundance, the treatment led to activation of 5′ adenosine monophosphate-activated protein kinase α (AMPKα) as reflected by increased phosphorylation of AMPKα at Thr-172 (Figure 3B). Once again, BAY-876 had no effect on ATP level or AMPKα phosphorylation in A2780 cells. Together, the results support the conclusion that BAY-876 impairs glycolysis-associated ATP generation via specific inhibition of GLUT1.

To further understand the metabolic outcomes of GLUT1 inhibition, we measured oxidative phosphorylation by following oxygen consumption rate (OCR) in SKOV-3, OVCAR-3 and A2780 cells treated with or without BAY-876. In contrast to the effect on glycolysis, BAY-876 increased OCR in these cells as determined using the Seahorse XF24 Analyzer (Figure 3C). Significant increases in basal mitochondrial respiration (basal OCR), ATP-linked OCR, and maximal respiratory capacity were observed in SKOV-3 cells. In OVCAR-3, increases in basal and ATP-linked OCR were detected. The mechanism leading to increased respiration following BAY-876 treatment was not fully understood. Since it was absent in A2780 cells, this effect of BAY-876 on OCR in SKOV-3 and OVCAR-3 cells was also GLUT1 dependent, likely as a compensatory response to inhibition of the GLUT1-glycolysis-ATP cascade.

### 2.4. Inhibition of GLUT1 Suppresses Proliferation, Viability, and Anchorage-Independent Growth of Ovarian Cancer Cells

**Figure 4:**
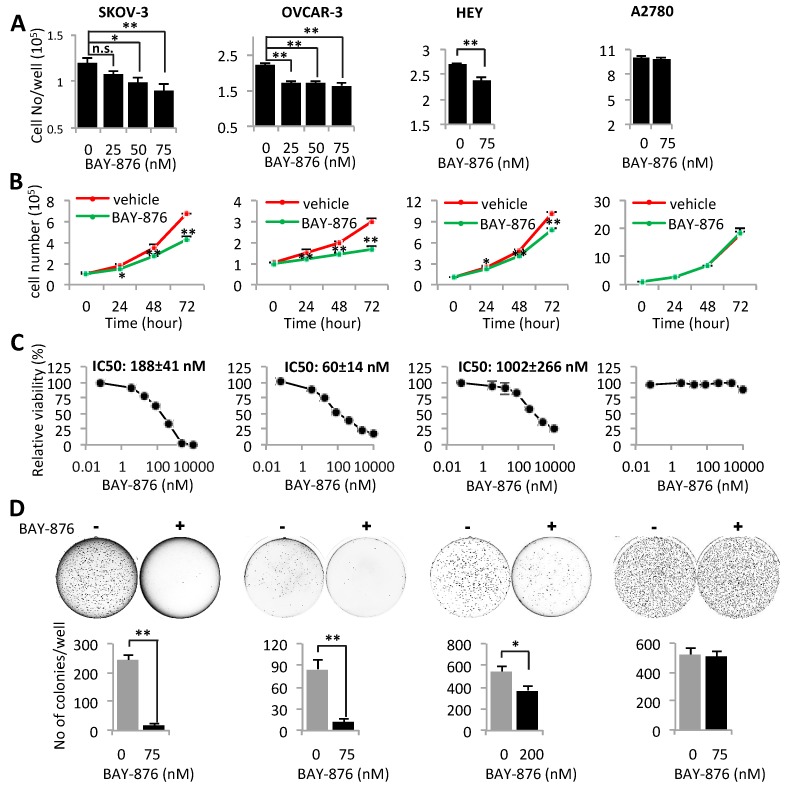
GLUT1 activity is crucial for cell growth. SKOV-3, OVCAR-3, HEY, and A2780 were cultured in 12-well plates with or without BAY-876 at indicated concentrations. The effects of BAY-876 on cell proliferation were analyzed by determination of cell numbers after 24 h (**A**) and 72-h growth curves (**B**). In (**C**), ovarian cancer cell lines were treated with indicated concentrations of BAY-876 for 72 h before the MTT assay to determine the cytotoxic effect of the compound. IC_50_ in each cell line was calculated using SigmaPlot 13.0 (Systat Software, San Jose, CA, USA). The effects of BAY-876 (75 nM for SKOV-3 and OVCAR-3, and 200 nM for the less sensitive HEY) on anchorage-independent growth were determined by soft agar assay (**D**). The data was presented as number of colonies per well. * *p* < 0.05; ** *p* < 0.01.

Given the importance of hyperactive glycolysis in support of the malignant features of cancer, we next assessed the effects of BAY-876 on growth and viability of ovarian cancer cells. First, we treated ovarian cancer cell lines with <100 nM concentrations of BAY-876 that significantly decreased glycolysis with little cytotoxicity as shown in Figure 1. Treatment with these concentrations of BAY-876 for a single day led to a dose-dependent decrease in numbers of SKOV-3 and OVCAR-3 cells (Figure 4A). Three-day growth curves in the presence of 75 nM BAY-876 further confirmed the growth-inhibitory effect in SKOV-3, OVCAR-3 and HEY, but not in A2780 cells (Figure 4B). To evaluate the combined effects of BAY-876 on cell growth and cytotoxicity, these cell lines were treated with a greater dose range of BAY-876 up to 10 µM for 3 days. The mitochondrial activity of viable cells was measured with the MTT assay. The results in Figure 4C revealed that OVCAR-3 was most sensitive to BAY-876 with IC_50_ value of approximately 60 nM. The IC_50_ values of SKOV-3 and HEY were 188 and 1002 nM, respectively. In agreement with the lack of functional GLUT1 and anti-glycolytic effect, A2780 cells were refractory to treatment with BAY-876 even at as high as 2 µM concentration (Figure 4C).

We also examined the ability of BAY-876 to inhibit anchorage-independent growth in soft agar, an in vitro analysis of tumorigenic potential of tumor cells in vivo. BAY-876 strongly inhibited formation of colonies in soft agar as indicated by decreased numbers and sizes of colonies in SKOV-3, OVCAR-3, and HEY, but not in A2780 cells (Figure 4D).

### 2.5. BAY-876 Inhibits Tumorigenicity of Ovarian Cancer Cell Lines and Ovarian Cancer PDXs

**Figure 5:**
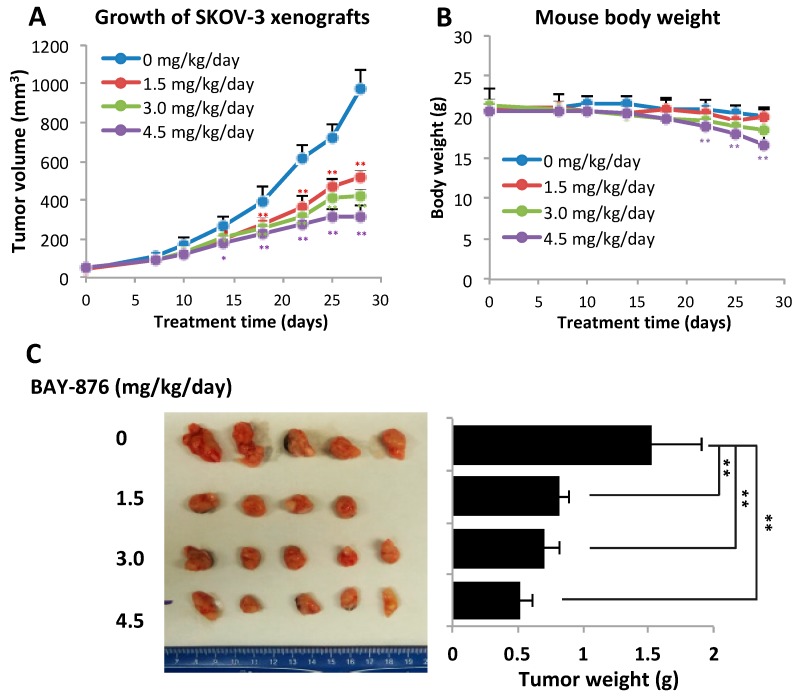
BAY-876 inhibits growth of SKOV-3 xenografts in mice. Four groups of female NSG mice bearing SKOV-3 xenografts were treated with BAY-876 for 28 days at dose of 0, 1.5, 3, or 4.5 mg/kg/day. Data shown were growth curves of tumor volumes (**A**), body weights over the course of experiment (**B**), images of end point tumors and final tumor weights (**C**). The data of all animal experiments were presented as mean ± SD. ** *p* < 0.01.

The roles of GLUT1 in regulation of glycolysis, energy metabolism, and anchorage-dependent and -independent growth of ovarian cancer cells suggest that GLUT1 is a promising anti-cancer target. Since BAY-876 has not been thoroughly evaluated as an anti-cancer agent in vivo, we assessed its anti-tumor potential and safety in female NOD-*scid IL2rg^null^* (NSG) mice carrying SKOV-3 subcutaneous (s.c.) xenografts. All animal experiments in this study were conducted following the policies and regulations of VCU IACUC. Four cohorts of mice with a similar range of tumor sizes (~100 mm^3^) were orally fed 0, 1.5, 3.0 and 4.5 mg/kg/day for 4 weeks. The tumor growth curves and changes in body weights were monitored and presented in Figure 5. There was a clear dose-dependent inhibition of tumorigenicity by BAY-876. The maximal effect was observed in the group treated with 4.5 mg/kg/day. The tumors shrunk significantly after 2 weeks of treatment. At endpoint, the final average tumor volumes and tumor weights decreased by 68% and 66%, respectively, compared to these parameters in the vehicle control group. However, the 4.5 mg/kg/day dose was somewhat toxic in NSG mice. The mouse body weights started to drop during the last week of treatment. At the end of 4 weeks, the weight loss in this group reached an average of 18% compared to the control group or groups of other two lower doses. Except for the weight loss, these mice, however, had no other noticeable health conditions.

**Figure 6:**
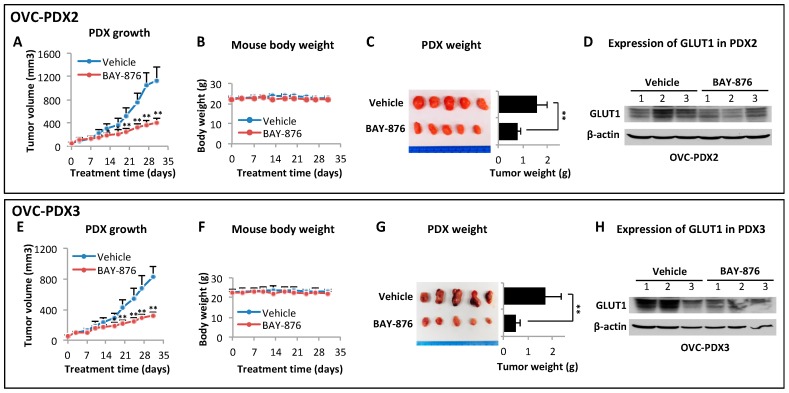
BAY-876 suppresses ovarian cancer PDXs in mice. Two serous ovarian carcinoma PDXs grown s.c. in female NSG mice were treated for 30 days with BAY-876 (4 mg/kg/day). Data shown were growth curves of tumor volumes (**A**,**E**), body weights over the course of experiment (**B**,**F**), images and weights of end point xenografts (**C**,**G**) and immunoblotting analysis of GLUT1 protein expression in PDXs from 3 vehicle- and 3 BAY-876-treated mice (**D**,**H**). ** *p* < 0.01.

We have developed PDXs from patients with high-grade serous ovarian carcinomas. H&E staining of these PDXs confirmed histological appearances of papillary adenocarcinomas, resembling the original patient tumor tissues (Supplementary Figure S2). We examined the effects of BAY-876 in two PDXs OVC-PDX2 and OVC-PDX3 that expressed GLUT1 protein (Figure 6D,H). Female NSG mice bearing PDXs were treated with 4.0 mg/kg/day, a dose determined on the basis of the earlier SKOV-3 xenograft experiment. Treatment with this dose of BAY-876 over 30 days dramatically decreased tumor growth with no significant loss of body weights (Figure 6B,F). The average endpoint tumor volumes reduced by more than 60% in both PDXs (Figure 6A,E). The final tumor weights in OVC-PDX2 and OVC-PDX3 were decreased by 50% and 71%, respectively (Figure 6C,G).

These results indicate that BAY-876 is an effective and safe anti-cancer agent when orally administrated at 4.0 mg/kg/day or less. We next asked whether acute treatment with a higher dose over a shorter period could achieve a better therapeutic benefit. Accordingly, female NSG mice carrying OVC-PDX2 were treated with 7.5 mg/kg/day and closely monitored for tumor growth and health conditions. Unfortunately, the mice did not tolerate this dose and all succumbed by 18 days of treatment. Shown in Supplementary Figure S3 were the effects of BAY-876 at 7.5 mg/kg/day on tumor growth, body weights, and Kaplan Meier survival of control and treated groups.

## 3. Discussion

In this study, we report that the recently developed GLUT1 inhibitor BAY-876 is a potent blocker of tumor-associated glycolysis and in vitro and in vivo growth of ovarian cancer cells. The target specificity of the compound is remarkably high as evidenced by nanomolar working concentrations, dependence of the activity on the presence of functional GLUT1 in responsive cells, and reproduction of the drug effect with molecular depletion of GLUT1.

The GLUT family consists of 14 members among which at least 11 are capable of transporting glucose under various experimental conditions [46], making it difficult to link a particular cellular or biochemical function to a specific GLUT isoform. Our results demonstrate that GLUT1, the isoform that is commonly upregulated in cancer [17], is a critical mediator of the glycolytic phenotype in ovarian cancer. Specifically, GLUT1 is required for maintaining high levels of basal glycolysis. The glycolytic activity of cancer is further upregulated in response to various stresses such as hypoxia, nutrient limitation or other detrimental factors associated with tumor microenvironment [47]. We showed that GLUT1 expression was induced in these conditions to further enhance glycolytic metabolism, a likely protective mechanism of tumor cells under adverse conditions. As a highly selective and potent inhibitor of GLUT1, BAY-876 suppressed both basal and induced glycolysis in ovarian cancer cells. Congruent with its glycolysis-targeting action in culture, oral administration of BAY-876 led to 50–71% reduction in tumorigenesis in both cell line- and ovarian cancer PDX models. Thus BAY-876 is an attractive candidate to be clinically tested as a glycolysis-targeted therapy in the future.

The reliance of tumor cells on elevated glycolysis for quick energy demands and uncontrolled proliferation has been known for almost one hundred years [3]. Although this signature of cancer cell metabolism has even been successfully exploited in FDG-PET diagnosis of tumors in clinical oncology [48], the effort in targeting glycolysis for treatment of cancer has not to date translated to clinical benefits. A number of challenges have impeded the progress in the field. First and most critically, our understanding of the mechanism underlying the glycolytic phenotype of cancer remains limited. Numerous glycolytic enzymes such as HK-2, PFK2, PKM2, LDHA, and PDK, have been reported to be rate-limiting factors that are dysregulated in various types of cancer [49]. Many of them are coordinately regulated by common signaling mechanisms in connection with activation of oncoproteins, loss of tumor suppressors or environmental changes [50]. Further adding to the complexity is the fact that each of these glycolytic enzymes has functional analogues. It is unclear how many players in the glycolytic pathway are involved in mediating active glycolysis in the malignant setting. An important question is whether targeting of a single node is sufficient or whether vertical intervention of multiple players along the pathway is necessary to significantly suppress tumor glycolytic rate and glycolysis-dependent growth. Furthermore, there are clearly diverse mechanisms implicated in the development of the glycolytic phenotype in different types of cancer [51]. It is therefore important to identify key glycolytic mediator(s) for therapeutic targeting in distinct types of cancer or in individual patients. Our study demonstrates for the first time a causal role for GLUT1 in maintaining glycolysis in high proportion of ovarian cancers. Although the results do not exclude existence of additional glycolytic players in ovarian cancer, inhibition of GLUT1 alone is sufficient to minimize glycolytic activity and growth of GLUT1-positive ovarian cancer cells.

In addition to elucidation of key glycolytic drivers, development of appropriate inhibitors against them is equally important to the success of glycolysis-targeted therapy. Several GLUT1 pharmacological inhibitors were reported prior to the discovery of BAY-876 [23,26,27,28,29,30,31]. None of them were considered to be of sufficient selectivity or potency against GLUT1. Their glycolysis inhibitory activities in cancer cells were either limited or have not been tested [23,26,28,29]. Our findings revealed that BAY-876 dose-dependently inhibited glycolysis. In most ovarian cancer cell lines we examined, 25 nM of BAY-876 rendered more than 50% inhibition of glycolysis. At similar concentrations, BAY-876 inhibited cell proliferation, suggesting that ovarian cancer cells rely on active glycolysis to fuel growth. A cytotoxic effect of BAY-876 was observed only at much higher concentrations. Consistent with these in vitro results, BAY-876 exerted a potent anti-tumor effect in both cell line- and ovarian cancer PDX models. At 4–4.5 mg/kg/day for 28–30 days, final tumor volumes and weights were decreased by 50–71%. Dose limiting toxicity was encountered at a higher concentration of BAY-876 administered to mice (Supplementary Figure S3). This is understandable given the known physiological role of GLUT1 in normal erythrocytes and endothelial cells of the blood-brain barrier [52]. Systemic toxicity is common with antitumor agents, not only those targeting the glycolysis pathway.

## 4. Materials and Methods

### 4.1. Reagents

BAY-876 was purchased from Chemgood (Glen Allen, VA, USA). The purity of a bulk order of one gram was confirmed to be higher than 99% with MRI. Radiolabeled D-[5-^3^H(N)]-glucose was obtained from Perkin Elmer (Boston, MA, USA). Metformin, etomoxir, oligomycin, carbonylcyanide-p-trifluoromethoxy-phenylhydrazone (FCCP), rotenone, and antimycin A were purchased from Sigma-Aldrich (St. Louis, MO, USA). MNS-B was prepared as we described previously [43]. Fetal bovine serum (FBS) was obtained from Atlanta Biological (Atlanta, GA, USA). All other cell culture reagents were obtained from Thermo Fisher Scientific (Waltham, MA, USA).

### 4.2. Data Mining

Gene expression data available on Oncomine (www.oncomine.org) were used for analysis of *GLUT1* mRNA levels in ovarian cancer and control normal ovarian tissues.

### 4.3. Cells

The ovarian cancer cell lines SKOV-3, OVCAR-3, HEY, A2780, and OVCA-429 were obtained originally from the American Type Culture Collection (ATCC, Manassas, VA, USA) or from Dr. G. Mills (MD Anderson Cancer Center). These cells were grown in RPMI 1640 supplemented with 10% FBS, 100 U/mL penicillin, and 100 µg/mL streptomycin. Cells were frozen at early passages and used for <10 weeks in continuous culture.

### 4.4. GLUT1 Knockdown

GLUT1 siRNA (Thermo Fisher Scientific, siRNA ID: s12925) or non-targeting control siRNA (Thermo Fisher Scientific, siRNA ID: 4390846) were transfected into SKOV-3 or OVCAR-3 cells using Amaxa™ Nucleofector™ Kit V according to the manufacturer (Lonza, Basel, Switzerland). One million cells were electroporated with 100 pmol siRNA. Twenty-four hours after plating, transfected cells were fed fresh medium and incubated for another 72 h before analysis of glycolysis and immunoblotting.

### 4.5. Glycolysis Assay

Glycolysis in cultured cells was measured as we described previously [39,40]. Briefly, cells were plated in 12-well plates and treated with indicated compounds. The cells were labeled with D-[5-^3^H(N)]-glucose (0.5 μCi/well) for the last 6 h. Hydrochloric acid was then added to the medium at a final concentration of 0.2 N to terminate all biological reactions. The acidified medium (0.6 mL) was collected into a 15-mL Falcon polypropylene conical tube (BD Biosciences, San Jose, CA, USA). A 0.5-mL microcentrifuge tube containing 0.25 mL of distilled water was uncapped and inserted into the 15-mL tube. The 15-mL tubes were tightly capped to allow diffusion between two liquid phases for 3 days. Radio-activities in the water phase and medium were determined by liquid scintillation counting. The conversion of D-[5-^3^H(N)]-glucose to ^3^H_2_O was calculated as we previously described [39,40] and glycolytic rate presented as % glucose conversion/5 × 10^5^ cells/6 h.

### 4.6. Lactate Production

Lactate levels in culture supernatants were measured with a colorimetric lactate quantification kit from Eton Bioscience (San Diego, CA, USA). The lactate concentrations of samples were normalized on 5 × 10^5^ cells.

### 4.7. Immunoblotting Analysis

Cells in culture or xenograft tissues were lysed with 1% Triton X-100-containing RIPA buffer supplemented with protease/phosphatase inhibitor cocktail from Roche Diagnostics (Indianapolis, IN, USA). Protein concentrations were quantified with the Pierce BCA Protein Assay Kit (Thermo Fisher Scientific). For analysis of GLUT1 (a membrane-associated hydrophobic protein), protein samples were solubilized in sodium dodecyl sulfate (SDS) Laemmli buffer and incubated at 65 °C for 10 min instead of boiling to prevent heat-induced aggregation of hydrophobic proteins. Equal amounts of proteins were resolved by sodium dodecyl sulfate polyacrylamide gel electrophoresis (SDS-PAGE). The antibodies against GLUT1 (catalog #12939), phospho-AMPKα (catalog # 2535), and β-actin (catalog # 4970) were purchased from Cell Signaling Technology (Danvers, MA, USA). The antibody for HIF-1α (catalog # 610958) was from BD Biosciences (San Jose, CA, USA). Immunocomplexes were visualized by an enhanced chemiluminescence detection kit (Thermo Fisher Scientific) using horseradish peroxidase-conjugated secondary antibodies (Cell Signaling Technology).

### 4.8. ATP Measurement

Cells were washed twice with PBS and lysed for 10 min in ice-cold water containing 0.75% NP-40. After centrifugation at 16,000× *g* for 3 min, the supernatants were diluted 100 times with H_2_O for ATP measurement with an ATP bioluminescence assay kit (Thermo Fisher Scientific). Cellular ATP contents were presented as nanomoles (nMol) per million cells.

### 4.9. OCR Measurement

OCR (pMoles/min) of cultured cells treated with or without BAY-876 was measured using the Seahorse XF24 Extracellular Flux Analyzer (Agilent Technologies, Santa Clara, CA, USA) as we described previously [43]. Ovarian cancer cell lines were seeded in XF24 microplates and cultured for 18–30 h before switching to the seahorse assay medium supplemented with 10 mM glucose and 2 mM glutamine. Basal mitochondrial respiration, ATP-linked respiration, maximal respiratory capacity and reserve capacity were calculated as described previously [43].

### 4.10. Cell Growth Assays

To assess the effect of BAY-876 (0, 25, 50, 75 nM) on proliferation, cells in 12-well plates were counted with Coulter counter after trypsinization. To determine the cytotoxic effect of BAY-876, cells were treated with a wider range of indicated concentrations for 3 days followed by MTT staining (Thermo Fisher Scientific) and measuring the absorbance at 570 nm. The IC_50_ values were calculated using SigmaPlot 13.0.

Anchorage-independent growth was performed to assess the effect of BAY-876 on the ability of cells to grow in semi-solid soft agar. Six-well plates were pre-coated with 1.5 mL of 0.6% soft agar in complete medium. Cells suspended in 1.5 mL growth medium containing 0.3% soft agar were overlayered onto the pre-coated wells. Fresh complete medium containing 0.3% agar (1.5 mL) was added to the top every 3–5 days. The colonies larger than 100 μm (SKOV-3, HEY and A2780) or 50 μm (OVCAR-3) in diameter were counted after 3 weeks. After crystal violet staining of colonies, pictures of plates were taken using Bio-Rad ChemiDoc™ Imaging Systems (Hercules, CA, USA).

### 4.11. Immunohistochemistry (IHC) Staining

IHC staining for CA125 was conducted with a monoclonal anti-CA125 antibody (catalog # MA5-12425) (Thermo Fisher Scientific) using the VECTASTAIN Elite ABC Kit (Vector Laboratories, Burlingame, CA, USA) as described previously [53].

### 4.12. Development of Ovarian Cancer PDXs

Ovarian cancer PDXs were established from fresh surgical specimens of patients diagnosed with high-grade serous ovarian carcinomas at the Virginia Commonwealth University (VCU) Health Network. Anonymized specimens were acquired from VCU Massey Cancer Center (MCC) Tissue & Data Acquisition & Analysis Core (TDAAC) within an average cold ischemic time of 45 min of removal from patients. The primary tumor tissues were minced to 1–2 mm^3^ fragments and implanted s.c. onto the right flank of 6–8 week-old female NSG/SCID mice obtained from VCU MCC Cancer Mouse Models Core. Once tumors grew to approximately 1 cm^3^, the tissues were re-transplanted and propagated for experiments in additional mice. The tumor histology was confirmed and compared with original patient tumor tissues by a board certified pathologist (Dr. M. Idowu).

### 4.13. Analysis of Anti-Cancer Activity of BAY-876 in Mice

The above-described PDXs and cell-line-derived xenografts were utilized to assess the anti-tumor effect of BAY-876. The cell lines in exponential growth phase were trypsinized, washed twice with PBS and resuspended in the serum-free medium. SKOV-3 cells (4 × 10^6^) were injected s.c. on the right flank of 6–7 weeks old female NSG mice. The formation of s.c. tumors was monitored and measured with a digital caliper. The tumor volumes were calculated based on the formula *lw*^2^/2 where *l* is the length and *w* is the shortest width of the tumor. When PDXs or cell line-derived tumors reached an average volume of 100 mm^3^, the mice were divided into control and experimental groups (5 mice/group) with a similar range of tumor volumes. The mice were treated by gavage feeding with the indicated doses of BAY-876. All animal experiments were conducted in compliance with the policies and regulations of VCU IACUC.

### 4.14. Statistical Analysis

All numerical data were presented as mean ± SD of triplicate assays. The statistical significances were calculated using Student’s two-tail *t* test, where *p* < 0.05 was considered statistically significant. The statistical significances were indicated with * if *p* < 0.05 or ** if *p* < 0.01.

## 5. Conclusions

In conclusion, our findings provide direct evidence that GLUT1 is causally linked to the glycolytic phenotype in ovarian cancer. Selective targeting of GLUT1 with the newly developed candidate inhibitor BAY-876 is sufficient to suppress glycolytic metabolism and in vitro and in vivo growth of ovarian cancer. Therefore BAY-876 is an ideal glycolysis-targeted anti-cancer agent.

## Supplementary Materials

The following are available online at http://www.mdpi.com/2072-6694/11/1/33/s1. Figure S1: Overexpression and gene amplification of GLUT1 in ovarian cancer; Figure S2: Establishment and characterization of ovarian cancer PDXs; Figure S3: The effects of a higher dose of BAY-876 in mice.
