# Supplementary material for: Ovarian Cancer Relies on Glucose Transporter 1 to Fuel Glycolysis and Growth: Anti-Tumor Activity of BAY-876
Source: Cancers (Basel). 2018 Dec 31;11(1):33. doi: 10.3390/cancers11010033 (PMC6356953; doi:10.3390/cancers11010033)
Supplement: Supplementary file 1 [file cancers-11-00033-s001.pdf]

Supplementary Materials: Ovarian Cancer Relies on Glucose Transporter 1 to Fuel Glycolysis and Growth: Anti-tumor Activity of BAY-876

Yibao Ma, Wei Wang, Michael O. Idowu, Unsong Oh, Xiang-Yang Wang, Sarah M. Temkin and Xianjun Fang

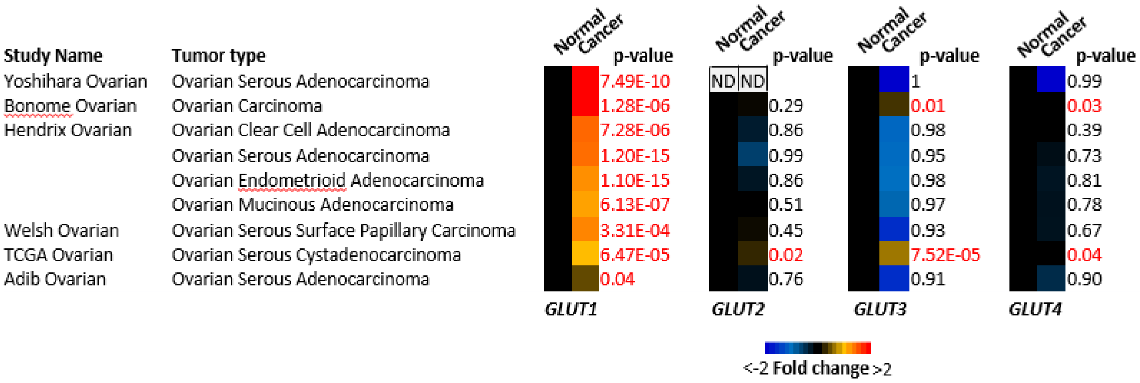

(**A**)

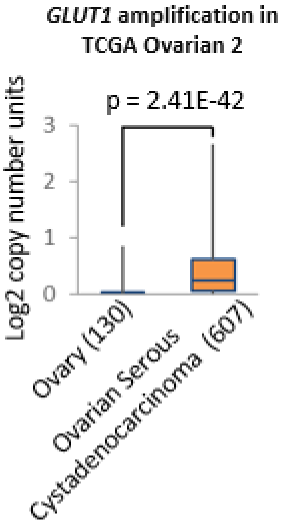

(**B**)

Figure S1. Overexpression and gene amplification of GLUT1 in ovarian cancer. (**A**)The publically available data on Oncomine were used to analyze *GLUT1-4* mRNA expression in ovarian cancer. Shown was heat map of fold changes in various histotypes of ovarian cancers versus normal ovaries. No data (ND) on *GLUT2* expression was available from the dataset of Yoshihara Ovarian. (**B**) Boxplot analysis of distribution rages of the relative *GLUT1* gene copies in the indicated numbers of normal ovaries and ovarian serous cystadenocarcinoma samples from the TCGA ovarian 2 dataset.

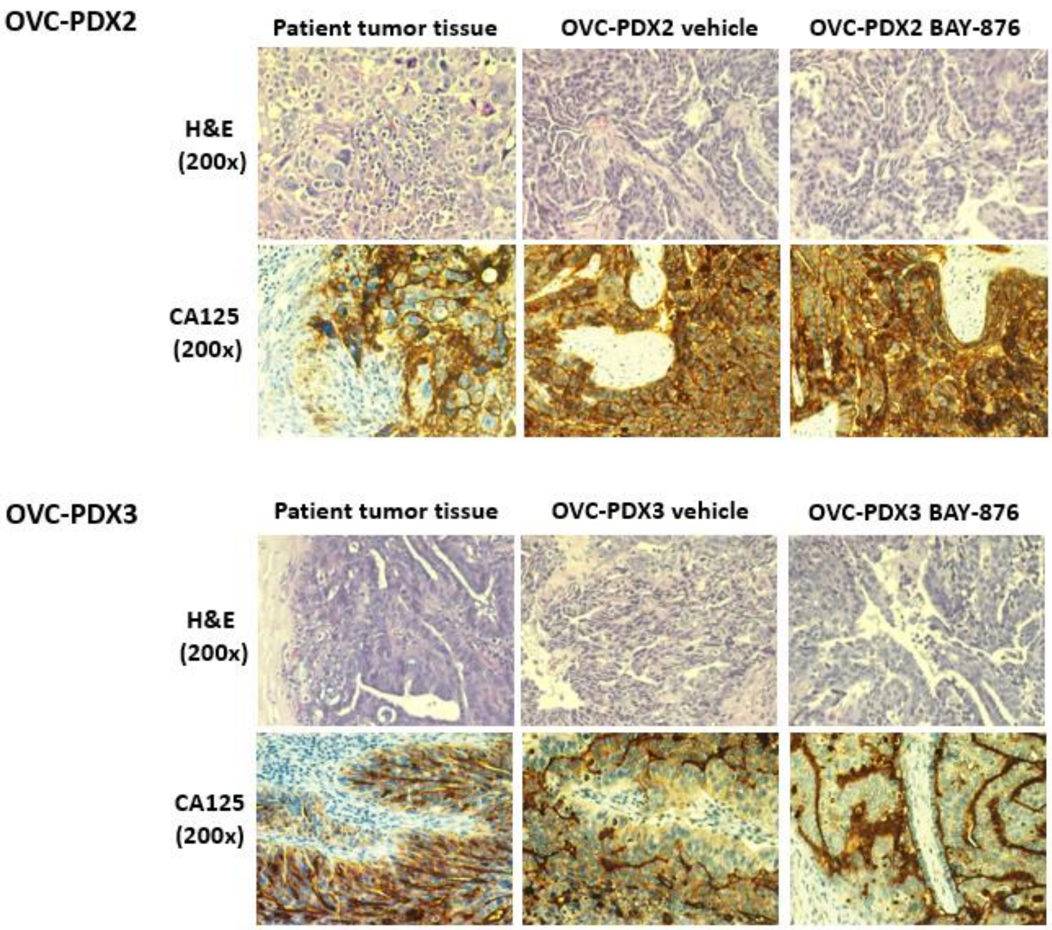

Figure S2. Establishment and characterization of ovarian cancer PDXs. Ovarian cancer PDXs were established s.c. in NSG female mice from patients diagnosed with serous ovarian carcinoma. H & E and IHC staining for CA125 were performed to verify histological appearances of serous ovarian carcinoma and expression of the ovarian cancer antigen CA125 in PDXs.

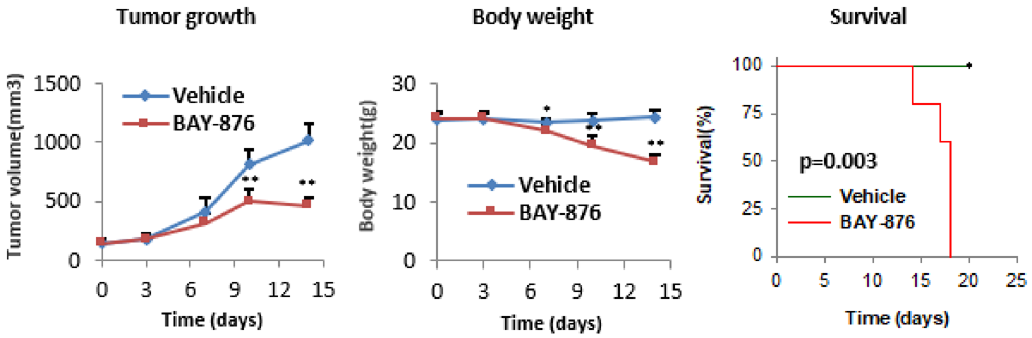

Figure S3. The effects of a higher dose of BAY-876 in mice. Female NSG mice bearing OVC-PDX2 were treated with vehicle or BAY-876 (7.5 mg/kg/day) for the indicated periods of time. Tumor volumes, body weights and mouse survival were monitored as detailed in Figure 8. Kaplan Meier survival and the LogRank test of two groups were performed using SigmaPlot 13.0.
